# Elevated Serum Sialic Acid Levels May be Associated With Diabetes Retinopathy: A Cross-Sectional Study in Ghana

**DOI:** 10.3389/fcdhc.2022.871051

**Published:** 2022-07-01

**Authors:** William K. B. A Owiredu, Christian Obirikorang, Alberta Boye Agoe, Emmanuel Acheampong, Enoch Odame Anto, Seth D. Amanquah, Hope Agbodzakey, Evans Asamoah Adu, Hubert Owusu

**Affiliations:** ^1^Department of Molecular Medicine, School of Medicine and Dentistry, Kwame Nkrumah University of Science and Technology (KNUST), Kumasi, Ghana; ^2^Medical Laboratory Unit, Greater Accra Regional Hospital, Accra, Ghana; ^3^Centre for Precision Health, School of Medical and Health Sciences, Edith Cowan University, Joondalup, WA, Australia; ^4^Department of Medical Diagnostics, Faculty of Allied Health Sciences, College of Health Sciences, Kwame Nkrumah University of Science and Technology, Kumasi, Ghana; ^5^Department of Chemical Pathology, University of Ghana Medical School, Korle-Bu, Accra, Ghana

**Keywords:** type 2 diabetes, micro-vascular complications, nephropathy, retinopathy, sialic acid, metabolic risk factors

## Abstract

This study determined the association between serum sialic acid (SSA) and metabolic risk factors in Ghanaian Type 2 diabetes (T2DM) with and without micro vascular complications. This cross-sectional study recruited 150 T2DM out-patients visiting the diabetic Clinic at the Tema General Hospital, Ghana. Fasting blood samples were collected and analyzed for Total Cholesterol (TC), Triglyceride (TG), Low Density Lipoprotein Cholesterol (LDL-C), High Density Lipoprotein Cholesterol (HDL-C), Fasting Plasma Glucose (FPG), Glycated Haemoglobin (HbA1c), SSA and C-Reactive Protein. SSA levels were significantly higher in diabetics with retinopathy (210.12 ± 85.09mg/dl) compared with those with nephropathy and those without complication (p-value= 0.005). Body adiposity index (BAI) (r= -0.419, p-value = 0.037) and Triglyceride (r= -0.576, p-value = 0.003), had a moderate negative correlation with SSA levels. In a One-Way Analysis of Covariance (Adjusted for TG and BAI), SSA could distinguish between diabetics with retinopathy and those without complications (p-value = 0.004) but not nephropathy (p-value = 0.099). Within group linear regression analysis showed that Elevated serum sialic acid was found in type 2 diabetic patients with retinopathic micro-vascular complications. Therefore, estimation of sialic acid levels may help with the early prediction and prevention of microvascular complications occurring due to diabetes, thereby decreasing the mortality and morbidity.

## Introduction

Type 2 diabetes is a chronic metabolic disorder characterized by persistent hyperglycemia (1). It has been associated with increased risk of cardiovascular comorbidities and microvascular complications including nephropathy, retinopathy and neuropathy (2). Diabetes-related nephropathy and retinopathy are common causes of chronic kidney diseases and non-congenital blindness worldwide (3). However, knowledge of this among Ghanaians remain extremely low with only 17.7% and 5.4% of diabetics having knowledge about such microvascular complications as retinopathy and nephropathy respectively (4). With a diabetes prevalence of 6%, higher than the continental average of 4.7% (5, 6), diabetes and its complications present an enormous socioeconomic and health burden for people in developing countries (7). Therefore, with the development and severity of diabetic complications being dependent on the duration of the disease and how early it is detected and managed (8), there’s then the need to identify early markers, which will not only monitor disease progression and prognosis soon after diagnosis, but also predict the onset of micro-vascular complications.

Sialic acid, also referred to as N-acetyl neuraminic acid, comprises the terminal component of oligosaccharide chains of several glycoproteins and glycolipids (9). Serum sialic acid concentration is a marker of the acute phase response and constitutes the terminal component of many acute phase proteins including α1-acid glycoprotein, haptoglobin, fibrinogen, transferrin and complement (10). Levels of serum sialic acid are increased in several pathologic conditions such as inflammation and malignancy (11). A cytokine-induced acute phase response has been hypothesized to play an integral role in the pathophysiology of Type 2 diabetes mellitus (T2DM) (12). Elevated levels of sialic acid predict individual features of metabolic syndrome such as hypertension and dyslipidaemia independently of body mass index (BMI) (13). This study was therefore focused on determining the association between sialic acid and known metabolic risk factors of T2DM in microvascular complications.

## Materials and Methods

### Study Design/Site

This cross-sectional study was conducted at the Diabetic Clinic, Eye Clinic and the Chemical Pathology unit of the Tema General Hospital. Tema General Hospital is the largest public health institution in the Tema Metropolitan area in Ghana. This area has a total projected population of 403,943. The hospital serves as a main referral centre within the Tema Metropolis. Its catchment area covers the whole metropolis including satellite towns and villages that extends as far as Sakumono, Lashibi and Nungua. It has twelve wards with a 294-bed capacity and also provides 24-hour Specialist and General Service to both in-patients and out-patients.

### Study Population

**Figure 1:**
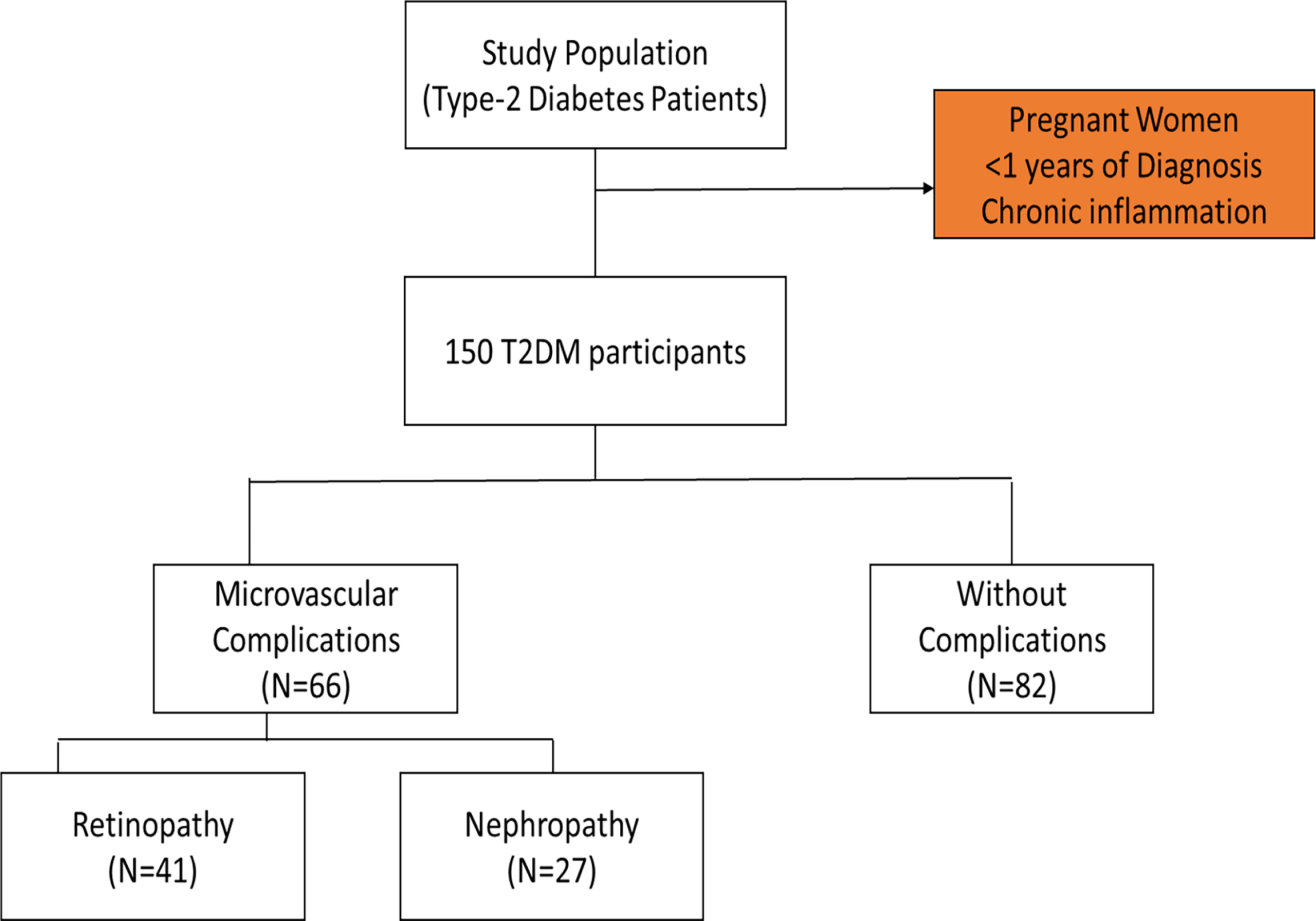
Flow diagram of the participant selection process.

The convenience sampling technique was used to recruit diabetic subjects scheduled for appointment at the diabetic outpatient’s clinic of the Tema General hospital during the study period. Participants included in the study comprised those who have been diagnosed of T2DM and were 40 years and above. The participants have had the condition for more than a year and were on diet with oral hypoglycaemic drugs. Pregnant women and participants with chronic inflammation from other infection were excluded. Out of the total of one hundred and fifty (150) T2DM patients recruited for the study, forty-one (41) were clinically diagnosed of diabetic nephropathy, twenty-seven (27) had been clinically diagnosed of retinopathy and eighty-two (82) had no complications (**Figure 1**). Pre-validated standard questionnaires were used to obtain socio-demographic and clinical information from the participants.

### Anthropometric Measurement

Body weight and height of study participants were measured using a standard physician’s scale and a stadiometer. Waist and hip circumferences were obtained with tape measure. Body mass index (BMI) was calculated as body weight (in kilograms) divided by the square of height (in meters). Waist-hip-ratio (WHR) and was calculated by dividing the waist circumference by the hip circumference. The body adiposity index (BAI) was calculated according to the formula as described by Bergman et al. (14), and visceral adiposity index (VAI) was calculated by the formula as described by Amato & Giordano (15).

### Blood Pressure Measurement

Blood Pressure (BP) was recorded after subjects had relaxed for at least 5 minutes. Measurements were taken with the subject being in the seated position using an automated BP monitor (Omron HEM-5001, Kyoto, Japan) placed on the subject’s right arm. Measurement was done twice within an interval of five minutes, and the average reading was recorded.

### Sample Collection and Processing

Venous blood samples (4 mls) were taken from subjects after 8-12-hour overnight fast. Two millilitres (2ml) each was placed in a Sodium fluoride and serum separator tubes. The samples in the Sodium fluoride tubes were centrifuged at 1,000g for 5 minutes and was used for the analysis of plasma glucose. Samples collected into the serum separator tubes were centrifuged at 1,000g for 15 minutes at room temperature after 30 minute-standing. Serum was separated into plain sample containers and frozen at -20°C for a period of up to one month until analysed.

### Biochemical Assay

Serum glucose, HbA1c, Total Cholesterol, Triglyceride and HDL-cholesterol were determined using BT 3000 Chemistry Auto Analyzer and reagent kits. The LDL cholesterol was derived Friedewald’s formula.

### Sialic Acid Assay

The double-antibody sandwich enzyme-linked immunosorbent one-step process assay (ELISA) was used to assay the level of lipid –bound sialic acid (LSA) in the samples. The standard, test sample and HRP-labelled LSA antibodies were added to enzyme pre-coated wells. These were incubated at 37°C for 60 minutes, washed to remove uncombined enzyme after which chromogen solutions were added. A colour change from blue to yellow after the acid reaction indicated a positive sample and absorbance measured at 450nm wavelength.

### Ozotex-C-Reactive Protein Determination

C-reactive protein was determined using the latex agglutination method. Serum samples were serially diluted and one drop of each diluted serum sample was placed in a glass slide circle. The content of each slide was mixed separately and spread with the mixing sticks provided in the kit. Agglutination within two minutes is a positive test and indicated the presence of CRP in the test specimen. The highest dilution that showed clear cut agglutination within 2 minutes indicated the CRP titre and the approximate concentration was obtained by multiplying titre by the sensitivity of the test.


$$\text{CRP in mg/dl }=\;\frac{\text{Highest dilution showing clear agglutination }(\text{D})}{\text{Sensitivity of the test }(\text{S})}$$

Where S= 0.6mg/dl.

### Ethical Consideration

The research protocol was reviewed and approved by the Committee for Human Research, Publications and Ethics (CHRPE) of the School of Medicine and Dentistry, KNUST (Ref no. CHPRE/AP/205/16) and the management of the Tema General Hospital. The study was conducted according to the guidelines of the Declaration of Helsinki.

The objectives and benefits of the study were explained to the diabetic patients at the time of initial data collection, and verbal and written consent were obtained from them. Respondents were assured that the information gathered was to be used strictly for research and academic purpose only. In addition, respondents were given the freedom to opt out any time they think they cannot continue with the study

### Statistical Analysis

Results were expressed as mean ± S.D. except where otherwise stated. Statistical analysis was performed using SPSS version 20.0 (SPSS Inc.) and GraphPad prism 5 for Windows. Normal distribution and homogeneity of the variances were tested using Kolmogorov-Smirnov and Levène tests, respectively. Student t-test was used to compare the significance of the difference in the mean values of any two groups and chi-square analysis was used to compare frequency between the two groups. One-way Analysis of variance/covariance and *post-hoc* with Bonferroni corrections were used evaluate the differences in mean SSA levels between the groups. Correlations between parameters were analysed using the Pearson r test for variables with normal distribution. Linear regression analysis was performed to evaluate the relationship between SSA and metabolic analytes within groups. P<0.05 was considered statistically significant.

## Results

Diabetic nephropathy and retinopathy were more prevalent in the female diabetics (68.3%, 66.7% respectively) than the male diabetics (31.7%, 33.3% respectively). However, while most risk factor parameters such as fasting blood glucose, HbA1c, blood pressure (SBP/DBP), and serum inflammatory markers did not show any statistically significant difference between the sexes, female diabetics also reported significantly lower levels of HDL-C (*P=*0.018), and significantly higher levels of BMI, VAI and BAI (P = 0.021, 0.001, and 0.007 respectively) (**Table 1**).

**Table 1:** Demographic and clinical parameters of study participants as stratified by gender.

Variable	Total (n=150)	Male (n = 58)	Female (n = 92)	P-value
**Age (Mean ± SD)**	58.90 ± 12.43	59.17 ± 13.72	58.73 ± 11.63	0.823
**Age group n (%)**				0.197
<30	3 (2.0)	3 (100.0)	0 (0.0)	
30-39	8 (5.3)	2 (25.0)	6 (75.0)	
40-49	25 (16.7)	8 (32.0)	17 (68.0)	
50-59	38 (25.3)	11 (28.9)	27 (71.1)	
60-69	44 (29.3)	20 (45.5)	24 (54.5)	
70-79	27 (18.0)	12 (44.4)	15 (55.6)	
≥ 80	5 (3.3)	2 (40.0)	3 (60.0)	
**WC (cm)**		91.59 ± 12.77	92.97 ± 13.62	0.539
**WHR**	0.54 ± 0.12	0.90 ± 0.08	0.91 ± 0.07	0.611
**Adiposity indices**				
**VAI**	1.72 ± 2.44	1.35 ± 0.09	1.96 ± 0.32	0.132
**BAI**	32.57 ± 7.93	29.89 ± 7.34	34.31 ± 7.82	**0.001**
**BMI n (Kg/m2)**		26.98 ± 5.20	29.54 ± 5.88	**0.007**
**BMI n (%)**				**0.021**
Underweight	3 (100)	1 (33.3)	2 (66.7)	
Normal	40 (100)	23 (57.5)	17 (42.5)	
Overweight	53 (100)	20 (37.7)	33 (62.3)	
Obese	54 (100)	14 (25.9)	40 (74.1)	
**Disease complication n (%)**				0.348
None	72 (100)	36 (43.9)	46 (56.1)	
Nephropathy	41 (100)	13 (31.7)	28 (68.3)	
Retinopathy	27 (100)	9 (33.3)	18 (66.7)	
**FBG (mmol/l)**	9.36 ± 3.82	9.39 ± 4.45	9.33 ± 3.40	0.929
**HBA1c (%)**	7.46 ± 1.38	7.21 ± 1.32	7.61 ± 1.40	0.078
**Blood Pressure (mmHg)**				
SBP	128.10 ± 17.08	127.50 ± 16.99	128.48 ± 17.22	0.734
DBP	81.67 ± 8.44	81.57 ± 8.67	81.74 ± 8.33	0.905
**Inflammatory parameters**				
SSA (mg/dL)	196.70 ± 87.21	192.00 ± 96.81	199.62 ± 80.99	0.604
CRP	0.12 ± 0.37	0.09 ± 0.04	0.13 ± 0.04	0.545
**Lipid profile**				
TC (mmol/L)	5.18 ± 1.31	4.93 ± 1.20	5.21 ± 1.30	0.191
TG (mmol/L)	1.20 ± 0.50	1.23 ± 0.65	1.17 ± 0.37	0.479
HDL-C (mmol/L)	1.38 ± 0.40	1.28 ± 0.34	1.44 ± 0.43	**0.018**
LDL-C (mmol/L)	3.56 ± 1.30	3.48 ± 1.26	3.61 ± 1.33	0.558

DBP, Diastolic blood pressure; SBP, Systolic blood pressure; FBG, Fasting blood glucose; HBA1c, glycated haemoglobin; SSA, Sialic acid; CRP, C-reactive proteins; WC, waistcircumference; WHR, Waist to hip ratio; VAI, Visceral adiposity index; BAI, body adiposity index; BMI, Body mass index; TC, Total cholesterol; TG, Triglyceride; HDL-C, High densitylipoprotein cholesterol; LDL-C, Low density lipoprotein cholesterol. The bold values indicates significance p-values.

SSA levels were elevated among all the diabetic study participants regardless of whether one was having any microvascular complications or not (**Table 2**). However, serum sialic acid was significantly elevated among diabetics with retinopathy compared with those with nephropathy, and without complications (p-value = 0.005). HbA1c levels differed among the three groups (p-value = 0.005; significantly lower among those with nephropathy but similar between retinopathy and without complications). Blood pressure showed a significant difference across the various groups. Waist circumference, waist-to-hip ratio, adiposity indices, CRP and BMI showed no statistically significant difference on comparison among the groups.

**Table 2:** Glycaemic indices, inflammatory markers, lipid profile, obesity and anthropometric indices among the T2DM patients stratified by disease complications.

Variables	Disease Complication			p-value
	None	Nephropathy	Retinopathy	
	(n = 82)	(n = 41)	(n = 27)	
**Age (Mean ± SD)**	57.22 ± 13.06	61.46 ± 11.53	60.11 ± 11.40	0.175
**FBG (mmol/l)**	9.25 ± 3.57	9.39 ± 4.12	9.62 ± 4.24	0.909
**HbA1c (%)**	7.60 ± 1.34	6.90 ± 0.97^*^^	7.89 ± 1.78	**0.005**
**Blood Pressure (mmHg)**				
SBP	126.89 ± 14.37	133.90 ± 23.01	122.96 ± 11.37**^#^**	**0.022**
DBP	83.11 ± 6.22	81.22 ± 11.22	78.00 ± 8.52**^#^**	**0.021**
***Inflammatory parameters***				
SSA (mg/dL)	172.22 ± 35.88	183.27 ± 39.1	210.12 ± 85.09^#^^	**0.005**
CRP	0.16 ± 0.05	0.09 ± 0.03	0.02 ± 0.02	0.197
***Lipid profile***				
TC (mmol/L)	5.05 ± 1.19	5.07 ± 1.46	5.31 ± 1.17	0.642
TG (mmol/L)	1.22 ± 0.57	1.15 ± 0.36	1.20 ± 0.42	0.799
HDL-C (mmol/L)	1.38 ± 0.41	1.28 ± 0.40	1.50 ± 0.35	0.076
LDL-C (mmol/L)	3.47 ± 1.26	3.75 ± 1.46	3.52 ± 1.16	0.527
***WC (cm)***	92.56 ± 15.46	90.59 ± 10.34	94.87 ± 9.42	0.428
***WHR***	0.90 ± 0.07	0.89 ± 0.08	0.92 ± 0.05	0.311
***Adiposity indices***				
VAI	1.52 ± 0.81	2.27 ± 0.48	1.53 ± 0.75	0.251
BAI	32.64 ± 9.77	32.01 ± 5.20	33.37 ± 4.30	0.788
BMI n (Kg/m2)	28.90 ± 6.33	28.14 ± 4.89	28.09 ± 5.02	0.708

*Significantly different on comparison between None vs nephropathy group, ^#^significantly different on comparison between non vs retinopathy group, ^ significant different on comparison between retinopathy vs nephropathy group, at P < 0.05. DBP, Diastolic blood pressure; SBP, Systolic blood pressure; FBG, Fasting blood glucose; HbA1c, glycated haemoglobin; SSA, Sialic acid; CRP, C-reactive proteins. WC, waist circumference; WHR, Waist to hip ratio; VAI, Visceral adiposity index; BAI, body adiposity index; BMI, Body mass index; TC, Total cholesterol, TG, Triglyceride, HDL-C, High density lipoprotein cholesterol; LDL-C, Low density lipoprotein cholesterol. The bold values indicates significance p-values.

**Figure 2:**
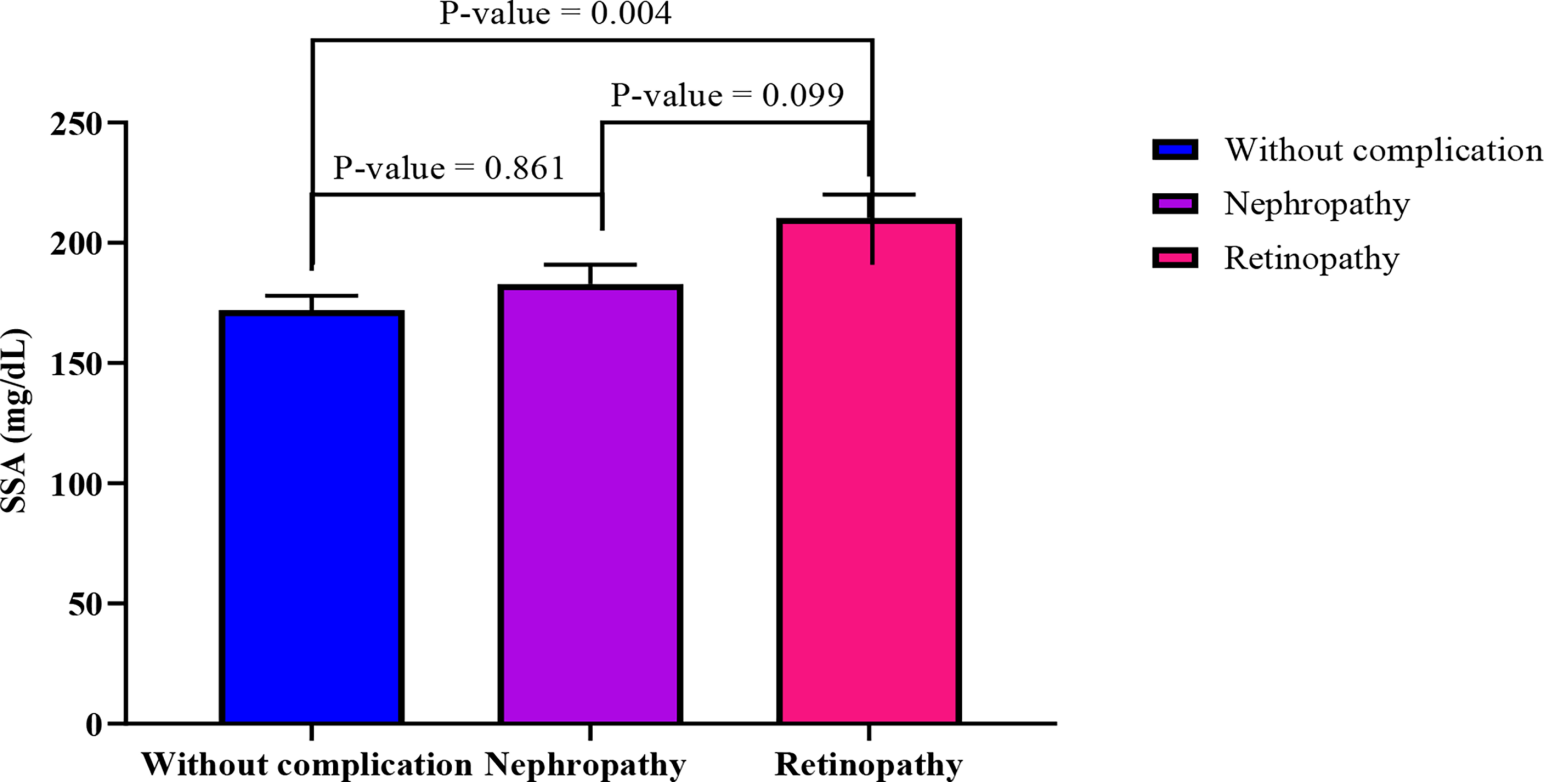
Comparison of estimated marginal means across the three groups using ANCOVA analysis adjusting for TG and BAI. The following values were obtained from the test of between subject model: F= 5.50, R^2^ = 0.079, p-value= 0.005, sum of squares = 38661.469.

When adjusted for age, increasing SSA levels shows a significantly moderate association with decreasing levels of triglycerides (r= -5.74, p-value = 0.003) among diabetics with retinopathy. Also, BAI showed moderate negative correlation with SSA (r= -0.419, p-value= 0.037) (**Table 3**). In an ANCOVA analysis adjusting for BAI and TG levels (**Figure 2**), mean SSA levels significantly differed between diabetics with retinopathy compared with those without complications (p-value =0.004).

**Table 3:** Partial correlation between sialic acid and metabolic risk factors among T2D with retinopathy, nephropathy, and those without microvascular complication controlling for age.

Variables		Sialic Acid (SSA)		
		Without Microvascular Complication (n=71)	Nephropathy (n=38)	Retinopathy (n=26)
**VAI**				
	r	0.069	0.105	-0.388
	p-value	0.578	0.535	0.055
**BAI**				
	r	0.044	0.313	-0.419
	p-value	0.722	0.059	**0.037**
**Systolic Pressure**				
	r	-0.089	0.232	0.317
	p-value	0.471	0.167	0.122
**Diastolic Pressure**				
	r	-0.148	0.124	0.333
	p-value	0.227	0.464	0.104
**Fasting Blood Sugar**				
	r	-0.165	-0.229	-0.231
	p-value	0.180	0.172	0.266
**HbA1c**				
	r	0.149	-0.235	-0.059
	p-value	0.226	0.161	0.779
**Total Cholesterol**				
	r	-0.061	0.216	-0.325
	p-value	0.62	0.198	0.113
**Triglyceride**				
	r	-0.043	0.248	-0.574
	p-value	0.727	0.139	**0.003**
**HDL**				
	r	-0.03	-0.123	-0.174
	p-value	0.806	0.469	0.405
**LDL**				
	r	-0.047	0.204	-0.326
	p-value	0.705	0.227	0.112
**C-Reactive Protein**				
	r	0.098	-0.099	0.225
	p-value	0.425	0.561	0.28

r, Pearson’s Correlation Coefficient; LDL-C, Low Density Lipoprotein cholesterol; HDL-C, High Density Lipoprotein cholesterol; HbA1c, Glycated Haemoglobin; VAI, Visceral Adiposity Index; BAI, Body Adiposity Index. The bold values indicates significance p-values.

A multiple linear regression analysis (stepwise) revealed that a one-point increase in Triglyceride level was associated with a significant decrease in SSA levels (-113.82, 95%CI: -181.91 to -45.74). Thus, variations in TG levels explains 33.2% of variations in SSA levels among diabetic patients with retinopathy, as shown by the R^2^ value in **Table 4**. However, these significant changes were not observed among diabetic patients without complications and those with nephropathy (p-value >0.05).

**Table 4:** Multiple regression analysis (Stepwise) to evaluate the relationship between serum sialic acid and metabolic analytes within the groups (retinopathy, nephropathy, and those without microvascular complication.

Case/Control	Model	R	Adjusted R Square	SEE	Change Statistics				
					R^2^change	F change	df1	df2	P-value
Without Microvascular Complication	a	.053a	-0.012	36.49804	0.003	0.186	1	67	0.667
	b	.060b	-0.027	36.7586	0.001	0.054	1	66	0.818
	c	.148c	-0.023	36.69853	0.018	1.216	1	65	0.274
Nephropathy	a	.242a	0.033	38.49679	0.059	2.243	1	36	0.143
	b	.358b	0.078	37.58126	0.069	2.775	1	35	0.105
	c	.358c	0.051	38.127	0.000	0.005	1	34	0.944
Retinopathy	a	.576a	0.304	71.005	0.332	11.905	1	24	**0.002**
	b	.660b	0.387	66.646	0.104	4.242	1	23	0.051
	c	.660c	0.359	68.144	0.000	0.000	1	22	0.999

a Predictors: (Constant), Triglyceride (Estimate = -113.82, 95%CI: -181.91 to -45.74).

b Predictors: (Constant), Triglyceride, BAI (Estimate: -6.43, 95%CI: -13.1- to 0.028).

c Predictors: (Constant), Triglyceride, BAI, Age (Estimate= 0.001, 95%CI: -2.45 to 2.26). The bold value indicates significance p-value.

## Discussion

Retinopathy and nephropathy are the major micro-vascular complications that lead to blindness and end-stage renal disease in diabetics (16). Many studies have indicated that diabetic complications are mainly due to the chronic hyperglycaemia that exerts its health effects through several mechanisms such as hypertension, dyslipidaemia, platelet activation, and altered endothelial metabolism (17). This study was therefore conducted to determine the association between sialic acid and known metabolic risk factors in Type 2 Diabetic patients with and without microvascular complications. Among the total number of patients recruited in this study, 68 presented with micro-vascular complications of which 39.7% had developed retinopathy and 60.3% had developed nephropathy. However, 54.7% of the diabetic patients did not have any microvascular complications.

There was an increasing trend in concentrations of serum sialic acid (SSA) levels among the diabetics in this study. Patients who had developed retinopathy had the highest level of SSA, followed by those who had developed nephropathy with the least levels seen in those without any microvascular complications. These concentrations of SSA in this study were higher than concentrations from diabetics in several other studies (18, 19) which found significantly elevated sialic acid levels in patients compared to their controls. These differences may be due to differences in methods used to measure SSA, the health status of the participants as well as individual differences in the SSA levels. In T2DM in general, the circulating sialic acid concentration is elevated in comparison with nondiabetic subjects (3). The vascular endothelium is enriched with sialic acid moieties which are released into circulation when there is extensive microvascular damage in T2DM. A cytokine-induced acute phase response exacerbated by the diabetic process has also been implicated to cause elevations in levels of SSA (20). This finding thus confirms that sialic acid may prove to be a useful marker in patients with T2DM particularly those with complications.

Diabetic patients with retinopathy had significantly higher SSA compared to those without any complications. This finding agrees with the study by Merat al (21)., and Crook et al. (22) but inconsistent with the study by Deepa et al. (23) who found no significant difference in serum sialic acid levels in diabetics with proliferative and non-proliferative retinopathy, non-retinopathic diabetics and non-diabetic patients. Again, unlike Prajna et al. (24) who observed significantly higher SSA among diabetics with nephropathy compared to patients without any complications, our results showed no significant difference between these category of diabetics. Khan et al. (25) in their study found significantly higher serum SSA levels in patients with retinopathy, nephropathy and coronary artery disease compared to diabetics who did not have complications. In this study, difference in SSA levels between patients who had developed retinopathy and those who had nephropathy did not reach statistical significance. Generally, sialic acid is bound to acute phase proteins with negligible free sialic acid in circulation. Thus, to explain further, the associations observed in this study, a study that evaluate free and bound SSA levels will be needed.

How, the likely explanation to these associations is that acute phase response and tissue injury caused by diabetic vascular complications is pronounced among patients with retinopathy. Sialic acid levels in plasma may be influenced by several factors including variations in the sialylation of apolipoproteins before their secretion into plasma; variations in the amount of sialic acid-containing apolipoproteins on lipoprotein in plasma; and modifications of the SSA on lipoprotein constituents following their secretion in plasma Crook et al. (22).

Hyperglycaemia is a significant stressor that has also been shown to cause chronic inflammation (12, 26). Elevated glucose levels could promote inflammation by increased oxidative stress (27), although the relationship between inflammation markers and glycaemic control is not been fully understood. SSA showed no significant relationship with HbA1c or FPG. This result is consistent with the findings of Lindberg et al. (28) who indicated that hyperglycaemia may be unlikely to have a major effect on the acute phase response in T2DM. Others studies (19, 24) have however found a significantly positive correlation between sialic acid, HbA1c and FPG.

Hypertension significantly impact the incidence and progression of cardiovascular events and microvascular complications (29). SBP was significantly higher in diabetic patients who had developed nephropathy than those with retinopathy in this current study. Aside CVDs, hypertension particularly magnifies risk of nephropathy which occurs in about 40% of diabetic patients (29). Population-based studies have shown that CVD mortality was 7.5 times greater among persons with T2DM and its risk was associated with elevated sialic acid levels (30, 31). Crook et al. (22) found significant association between total sialic acid and both SBP and DBP in their study although the relationship between the lipid-associated sialic acid levels and systolic pressure did not reach significance in T2DM. These findings are consistent with this study, where no significant association was observed between blood pressure and sialic acid.

Furthermore, there was no statistically significant difference in lipid parameters among the three categories of diabetic patients except for triglyceride levels, which showed a significant negative correlation with SSA levels among diabetics with retinopathy. Reports from other studies (32–34) have however indicated a significant correlation between sialic acid and TG, TC and LDL-C. Inconsistent with our findings, Crook et al. found no significant relationship between sialic acid and TG in diabetics with retinopathy (22).

Production of inflammatory mediators by visceral adipose tissue induces the release of acute-phase reactants in hepatocytes and endothelial cells (35). Elevated CRP levels have been associated with abdominal adiposity in some studies (36–40). Waist circumference, waist-to-hip ratio as well as BAI and VAI did not vary among the diabetic patients. However, while serum sialic acid showed a significantly inverse relationship with BAI among participants with retinopathy, it rather showed a non-significant positive correlation among those with nephropathy. In patients without any microvascular complications, SSA showed a significantly positive correlation with BAI.

The are some limitations which are vital when interpreting the findings of this study. First, the small sample size has a significant impact on the power, interpretation of the results and inconsistent findings with other studies. Second, the cross-sectional study design of the current study did not allow generalisation of our result in the general population, thus the utility of sialic acid estimation in early prediction and prevention of microvascular complication in diabetes. However, there were considerable number of studies that supported our study findings. Thus, a prospective cohort study with larger sample size, considering the measurement of bound and free sialic acid. in addition, to the routine analytes will be useful to evaluate this associations with precision.

## Conclusion

Elevated serum sialic acid was associated with the presence nephropathic and retinopathic micro-vascular complications in type 2 diabetic patients. There was also direct association of HbA1c with elevation of SSA and CRP.

## Data Availability Statement

The raw data supporting the conclusions of this article will be made available by the authors, without undue reservation.

## Ethics Statement

The studies involving human participants were reviewed and approved by Committee of Human Research, Publications and Ethics, School of Medicine and Dentistry, KNUST, Kumasi. The patients/participants provided their written informed consent to participate in this study.

## Author Contributions

WO, AB, and CO designed the study. Research data collection and laboratory analysis was performed by AB and HA. The data analysis and interpretation were performed by HA, EA, EOA, and EAA. SA, HA, and AB wrote the manuscript. WO, CO, SA, EOA, and EA reviewed the manuscript. All authors read and approved the final manuscript.

## Funding

This study did not receive funding from private, government or not-for-profit organization and was fully funded by authors.

## Conflict of Interest

The authors declare that the research was conducted in the absence of any commercial or financial relationships that could be construed as a potential conflict of interest.

## Publisher’s Note

All claims expressed in this article are solely those of the authors and do not necessarily represent those of their affiliated organizations, or those of the publisher, the editors and the reviewers. Any product that may be evaluated in this article, or claim that may be made by its manufacturer, is not guaranteed or endorsed by the publisher.
